# Efficacy of CTPV for Diagnostic and Therapeutic Assessment: Comparison with Endoscopy in Cirrhotic Patients with Gastroesophageal Varices

**DOI:** 10.1155/2020/6268570

**Published:** 2020-06-05

**Authors:** Zijin Cui, Haiqing Yang, Xiaoxu Jin, Huiqing Jiang, Wei Qi, Wenfeng Feng, Zhijie Feng

**Affiliations:** ^1^Gastroenterology, The Second Hospital of Hebei Medical University, Shijiazhuang, Hebei, China; ^2^Gastroenterology, Hebei General Hospital, Shijiazhuang, Hebei, China; ^3^Radiology, The Second Hospital of Hebei Medical University, Shijiazhuang, Hebei, China

## Abstract

**Background and Aims:**

Computed tomography portal venography (CTPV) shows potential in detecting varices that need treatment and their drainage pathways. However, its agreement with endoscopy requires further study. We investigated the feasibility of CTPV as an alternative tool to endoscopy in screening gastroesophageal varices (GEVs) and developed a CTPV-based model to provide a less invasive assessment of endotherapy for cirrhotic patients with GEVs.

**Methods:**

The study included 33 cirrhotic patients with a recent history of variceal hemorrhage. The presence, grade, and classification of GEVs on endoscopy and CTPV were compared (kappa test). Twenty-four patients were treated endoscopically, including 12 for esophageal varices (EVs), 8 for gastric varices (GVs), and 4 for GEVs. Treatment efficacies were assessed with the newly developed CTPV-based method at 1 week and 1 month after treatment. Efficiency evaluated by CTPV and endoscopy was compared by Fisher's exact test to determine whether CTPV is efficient in the assessment of endotherapy efficacy.

**Results:**

For the screening and grading/classification of EVs and GVs, substantial agreement (EV kappa: 0.63 and 0.68; GV kappa: 0.62 and 0.75, respectively) was noted between endoscopy and CTPV. The therapeutic efficacy of EVs was higher when assessed by CTPV than when evaluated by endoscopy (37.50% vs. 12.50% at 1 week postoperation, *P* = 0.22; 62.50% vs. 25.00% at 1 month postoperation, *P* = 0.07), but without statistical significance. The same trend was also found in the assessment of therapeutic efficacy for GVs (25.00% vs. 16.67% at 1 week postoperation, *P* = 1; 58.33% vs. 41.67% at 1 month postoperation, *P* = 0.68).

**Conclusion:**

CTPV is comparable to endoscopy in the detection of GEVs and in the evaluation of endotherapy efficacy, which suggests that it could be a less invasive alternative for endoscopy in cirrhotic patients with GEVs needing treatment.

## 1. Introduction

The development of gastroesophageal varices (GEVs) is a severe complication of cirrhosis, and their rupture is a substantial cause of death in cirrhotic patients [1]. Currently, the diagnosis and treatment of GEVs depend mainly on endoscopy [2]. Repeated endoscopy is needed after the initial management of GEVs to assess the endoscopic efficacy and decide the timing of retreatment. All of these procedures place a significant burden on patients due to discomfort and economic costs during repeated endoscopy. Thus, it is essential to find effective and less invasive alternatives. Computed tomography portal venography (CTPV) comprehensively depicts the portal venous system with three-dimensional reconstruction and, as a result, has been increasingly used in GEV patients [3]. The agreement between CTPV and endoscopy in GEV screening should be further identified [4]. In addition, studies have revealed that the prognosis of GEV patients is closely correlated with GEV volume and the diameter of its afferent vein after treatment, showing the evaluative potential of CTPV in endotherapy [5, 6]. The present study attempts to determine the agreement between CTPV and endoscopy in GEV detection and evaluate the efficacy of endotherapy using CTPV.

## 2. Patients and Methods

### 2.1. Patient Population

Patients were enrolled between December 2012 and August 2014. Eligible patients had a history of variceal hemorrhage secondary to cirrhosis within 3 months before hospitalization. Patients with portal vein thrombosis, severe ascites, or hepatocellular carcinoma or who experienced partial splenic embolization, splenectomy, and changed drug therapy during the follow-up period were excluded. This study was approved by the ethics committee at the Second Hospital of Hebei Medical University (ID: 2015070). Written informed consent was obtained from all subjects.

### 2.2. CTPV

All patients underwent CTPV with the absence of active bleeding for more than 48 hours after hospitalization. Before the examination, the patients fasted for 8–12 hours and practiced breathing exercises. Patients with tachycardia greater than 100 beats/min were given propranolol hydrochloride.

CTPV was performed using a 256-detector row CT scanner (Philips Brilliance iCT, Philips Healthcare, Best, The Netherlands) in three phases. Before the examination, 80 mL of iohexol (Omnipaque 350; Nycomed Amersham, Princeton, NJ) was administered intravenously as a contrast medium via a hand vein at 3.5 mL/s using a high-pressure injector. Images were obtained during the portal phase from the dome of the diaphragm to the iliac wing approximately 50–60 s after contrast injection, with the following parameters: section collimation, 0.625 mm; section thickness, 5 mm; pitch, 0.9; rotation time, 0.5 s per rotation; 120 kV; 343 mA. Three-dimensional CTPV images were reconstructed at an interval of 1.0 mm using the Philips EBW 4.5 workstation. Both maximum intensity projection and volume rendering were used for image reconstruction.

### 2.3. Image Analysis

Two radiologists who were blinded to the patients' clinical information evaluated both the two-dimensional transverse and 3D images independently. The images were reviewed for the presence of portosystemic collaterals and GEV drainage pathways.

The screening feasibility of CTPV for GEVs was evaluated by comparing the grades and classification of GEVs between CTPV and endoscopy. The types of gastric varices (GVs) were defined according to the Sarin classification system (Supplementary Method (available here)). Esophageal varices (EVs) were graded using a modified system proposed by Kim et al. (Supplementary Method). Two reviewers evaluated the EV grade and the GV type by consensus.

### 2.4. Endoscopy

Among 24 patients who received endoscopic treatments prophylactically, the treatment methods were selected according to the GEV characteristics and CTPV findings. Upper gastrointestinal endoscopy (GIF-HQ260 or GIF-HQ290, Olympus Tokyo, Japan; EG-590WR, Fujinon, Saitama, Japan) was performed within 8 days before or after CTPV examination. All endoscopic findings were captured as digital images and were reviewed independently by two experienced gastroenterologists who did not participate in the endoscopic examination and who were blinded to the CTPV findings. The reviewers recorded the varices within the digestive tract and determined the EV grade and GV type. EVs were graded according to the criteria proposed by the Chinese Society of Digestive Endoscopy (Supplementary Method), and GVs were classified using the same system as that used for CTPV images.

### 2.5. Efficacy of Endoscopic Treatments

Repeat CTPV and endoscopy were performed at a mean of 7 days and 30 days, respectively, after endoscopic treatment to evaluate changes in the varices, blockages of the afferent veins, and adverse events, including ectopic embolism and portal vein thrombosis. The patients were treated endoscopically until complete disappearance of the varices.

On both CTPV and endoscopy, the efficacy of esophageal endotherapy was assessed as follows: effective, a decrease of two grades after treatment; moderately effective, a decrease of one grade after treatment; and ineffective, no obvious change in the grade.

On CTPV, the efficacy of gastric endotherapy was assessed according to the decrease in the diameter of the feeding vessels and variceal volume. For nodular or pampiniform varices, the efficacy was assessed as follows: effective, ≥50% reduction in the cross-sectional area of the varices after treatment; moderately effective, 25–50% reduction after treatment; and ineffective, <25% reduction after treatment. For linear varices, the efficacy was assessed as follows: effective, ≥50% reduction in the maximum diameter of the varices after treatment; moderately effective, 25–50% reduction after treatment; and ineffective, <25% reduction after treatment.

On endoscopy, the efficacy of gastric endotherapy was subjectively assessed according to the decrease in the variceal volume as follows: effective, ≥50% reduction in the variceal volume after treatment; moderately effective, 25–50% reduction after treatment; and ineffective, <25% reduction after treatment.

### 2.6. Statistical Analysis

Kappa values were calculated to measure the agreement between endoscopy and CTPV in the grading and classification of GEVs. Kappa values > 0.81 were considered to indicate almost perfect agreement; 0.61–0.80, substantial agreement; 0.41–0.60, moderate agreement; and <0.40, fair agreement. Fisher's exact test was used for comparison of categorical data. A two-tailed *P* value less than 0.05 was regarded as statistically significant. Data analyses were performed using SPSS 19.0 (IBM Corp., Armonk, NY).

## 3. Results

### 3.1. Patient Characteristics

Thirty-three patients (age 50.70 ± 12.72 years, male 63.64%) met the enrollment criteria and were screened with CTPV and endoscopy. The etiology for cirrhosis was viral in 23 patients (HBV 17, HCV 6), primary biliary cirrhosis in 3, alcohol abuse in 2, drug induced in 1, and others in 4. The distribution of Child-Pugh scoring was 5 in A, 11 in B, and 17 in C. Nine patients were further excluded for the following reasons: 3 refused endoscopic intervention, 2 received treatment with *β* blockers right after endotherapy, and 4 received reexamination behind schedule. Finally, a total of 24 patients (age 50.92 ± 12.68 years, male 66.67%) were treated endoscopically, including 12 for EVs, 8 for GVs, and 4 for GEVs. At 1 week and 1 month after that, these 24 patients all underwent repeated endoscopy and CTPV (Supplementary Table 1).

### 3.2. Diagnostic Performance of CTPV for Varices within the Digestive Tract

**Figure 1:**
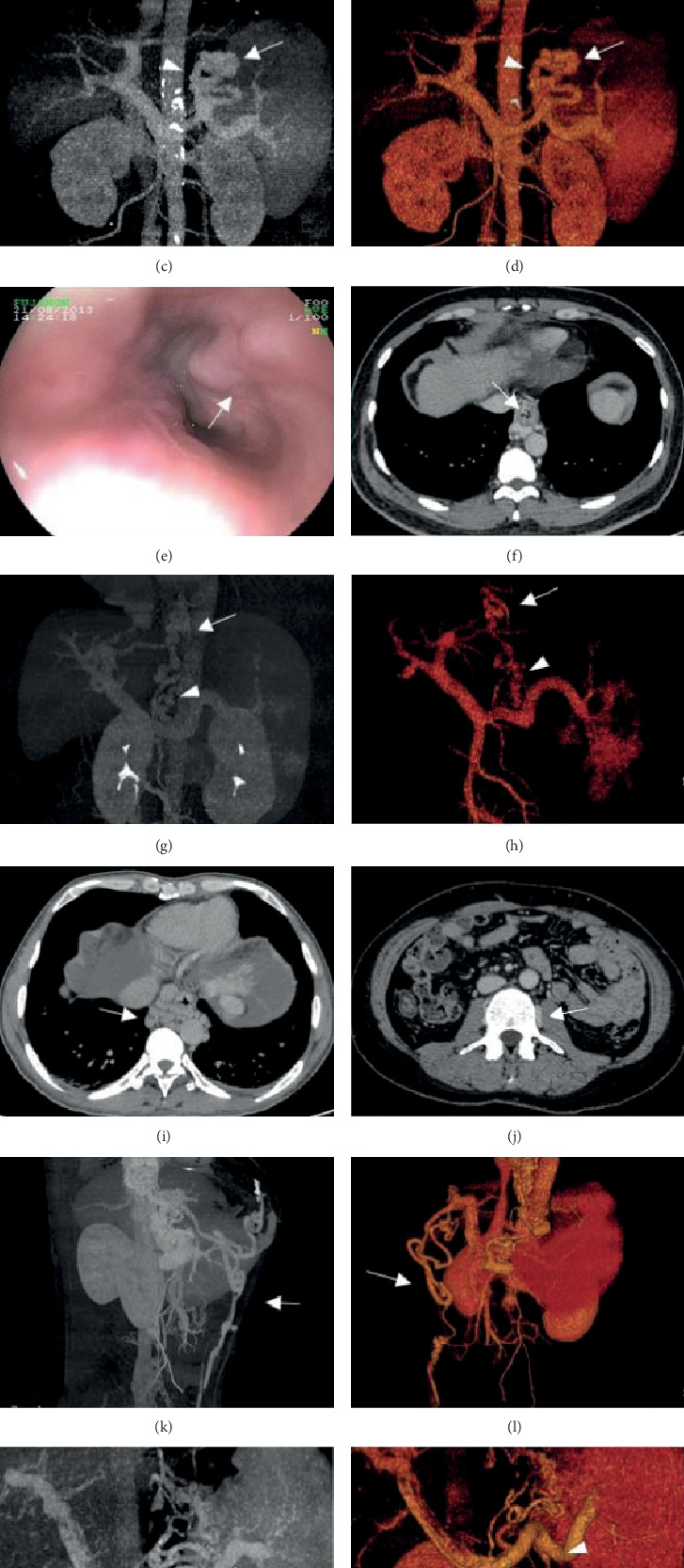
Computed tomography portal venography (CTPV) is comparable to endoscopy in the detection of gastroesophageal varices, and it is superior to endoscopy in the evaluation of the whole portal system. Severe gastric varices were clearly revealed by both (a) endoscopy and (b) CTPV. The (c) CT-MIP images and (d) CT-VR images demonstrate gastric varices (arrow) originating from the short/posterior gastric vein (arrowhead). Esophageal varices are depicted by (e) endoscopy and (f) CTPV. The (g) CT-MIP images and (h) CT-VR images reveal the left gastric vein as the inflowing vessel (arrowhead) of the varices (arrow). Axial computed tomography images show (i) paraesophageal varices and (j) paravertebral varices. Reconstructed images show the (k, l) tortuous paraumbilical vein and (arrow, m and n) spontaneous splenorenal shunt connecting the (curved arrow, m and n) left renal vein and (arrowhead, m and n) splenic vein.

Endoscopy and CTPV identified 30 EVs in 33 patients (90.91%). Additionally, one patient was diagnosed with EVs only by endoscopy, and another patient was diagnosed with EVs only by CTPV without endoscopic confirmation. The kappa value for the diagnostic agreement of EVs between endoscopy and CTPV was 0.63, indicating substantial agreement. Endoscopy identified 29 GVs in 33 patients (87.88%), and CTPV identified GVs in 28 (84.85%) of these patients. Additionally, in one patient with negative endoscopic results, GVs were identified on CTPV. The kappa value for the diagnostic agreement of GVs between endoscopy and CTPV was 0.62, indicating substantial agreement (Figure 1 and Supplementary Table 2).

Preoperatively on CTPV, EVs were found to be mild in 4 patients, moderate in 6 patients, and severe in 20 patients; on endoscopy, EVs were found to be mild in 3 patients, moderate in 7 patients, and severe in 20 patients. The kappa value was 0.68, indicating substantial agreement (Figure 1 and Table 1). Regarding the type of GVs, on CTPV, type one gastroesophageal varices (GOV1) were noted in 15 patients, GOV2 in 16 patients, and isolated GV (IGV1) in one patient; on endoscopy, GOV1 was noted in 17 patients, GOV2 in 14 patients, and IGV1 in two patients. The kappa value was 0.75, indicating substantial agreement (Figure 1 and Table 2). Duodenal varices were noted in one patient (3.03%) on both CTPV and endoscopy (Table 3).

### 3.3. Extraluminal CTPV Findings

Among the 33 patients with GEVs, the afferent vessel was the left gastric vein in 13 (39.39%), posterior gastric vein/short gastric vein in 4 (12.12%), and left gastric vein+posterior gastric vein/short gastric vein in 16 (48.48%); the efferent vessel was the azygos vein in 15 (45.46%), gastrorenal shunts in 5 (15.15%), and azygos vein+gastrorenal shunts in 13 (39.39%).

In addition, 21 paraesophageal varices (63.64%), 5 abdominal wall varices (15.15%), and 2 paravertebral varices (6.06%) were found on CTPV (Figure 1 and Table 3).

### 3.4. Efficacy of Treatments for EVs Evaluated by CTPV and Endoscopy

**Figure 2:**
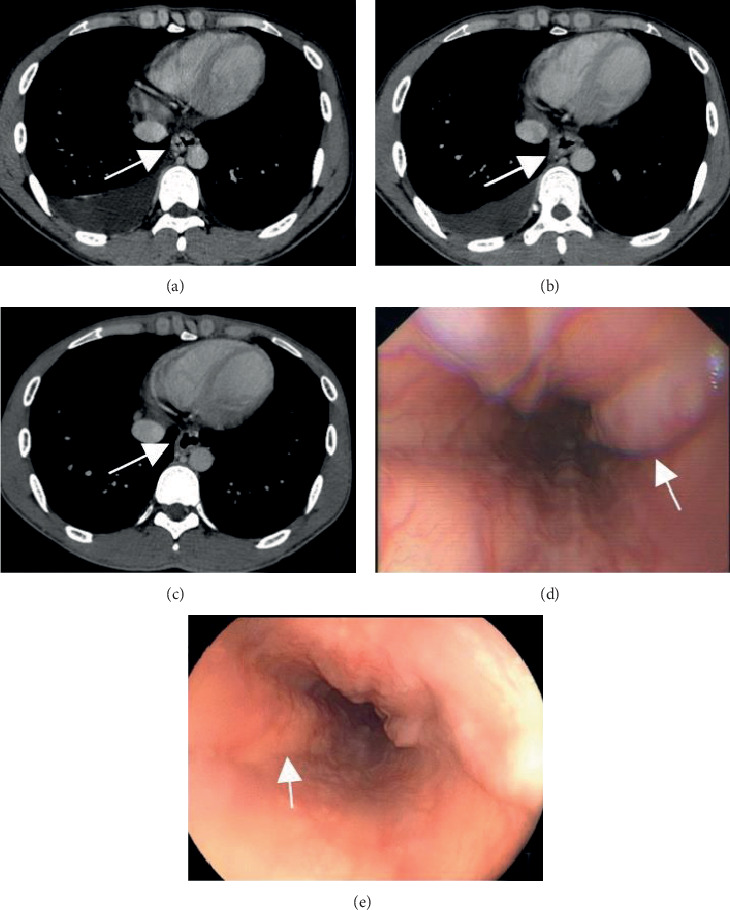
Efficacy of endotherapy in a patient with severe esophageal varices. Axial computed tomography images show that the (a) esophageal varices are attenuated with endoscopic band ligation at (b) 1 week and (c) 1 month after treatment. The (d) dilated varices are reduced at (e) 1 month after treatment on endoscopy.

Sixteen EVs were treated endoscopically. On CTPV, esophageal endotherapy was moderately effective in 6 cases at 1 week after treatment, with an overall effective rate of 37.50%. Additionally, the procedure was effective in 2 cases and moderately effective in 8 patients at 1 month after treatment, with an overall effective rate of 62.50%. On endoscopy, esophageal endotherapy was moderately effective in 2 cases at 1 week after treatment, with an overall effective rate of 12.50%. Additionally, the procedure was effective in 1 case and moderately effective in 3 cases at 1 month after treatment, with an overall effective rate of 25.00%. No significant difference was found between CTPV and endoscopy in the therapeutic evaluation of EVs (Figure 2 and Table 4).

### 3.5. Efficacy of Treatments for GVs Evaluated by CTPV and Endoscopy

**Figure 3:**
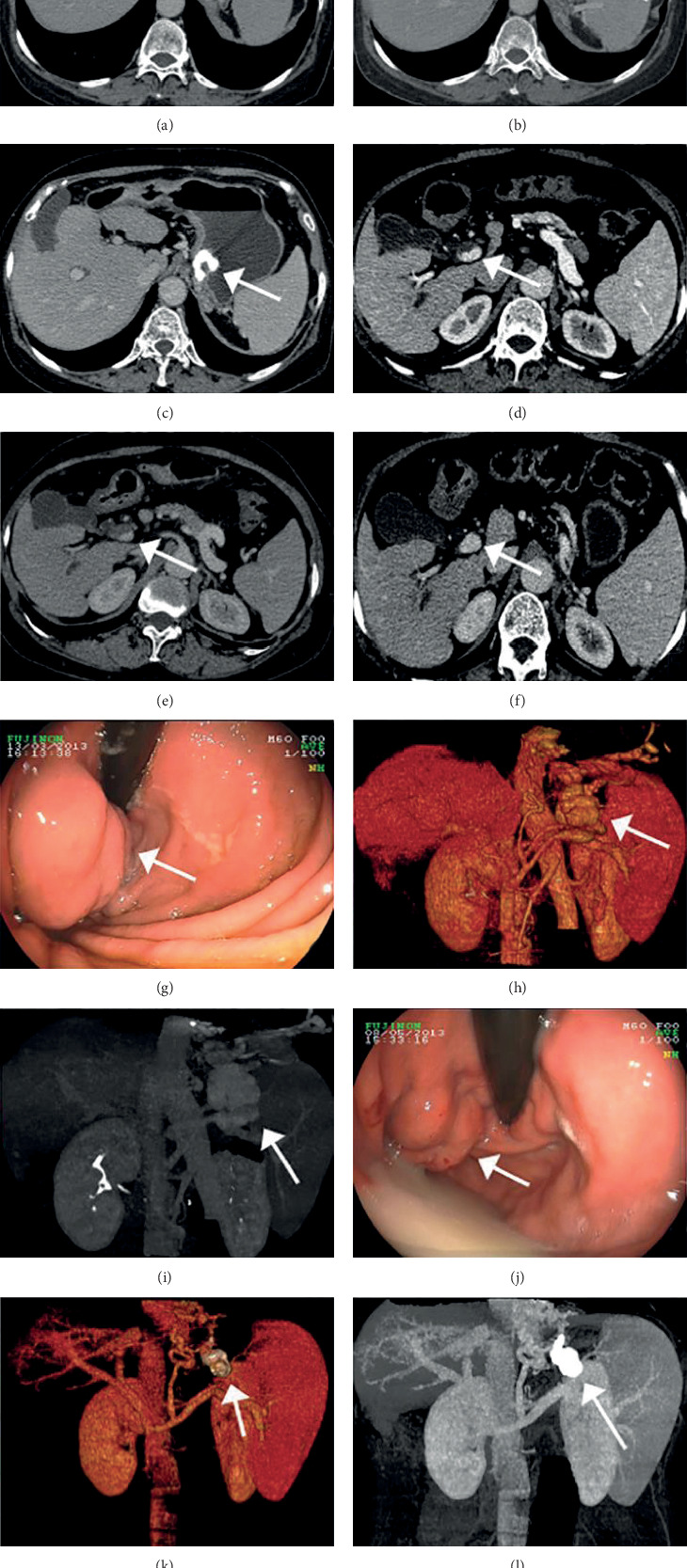
Efficacy of endotherapy in a patient with severe gastric varices. Axial computed tomography images show (a) nodular gastric varices located in the fundus. (b) One week after tissue adhesive injection, the afferent vein is partly embolized (arrowhead). (c) One month later. (d) The patient experienced portal vein thrombosis before treatment. (e) The filling defect of the portal vein was aggravated 1 week after treatment, and (f) it improved 1 month after treatment. (g, j) Endoscopic images show the changes in the varices 1 month after treatment. The reduced volumes of the gastric varices are shown with (h, k) volume rendering and (i, l) maximum intensity projection three-dimensionally.

Twelve cases with GVs were treated endoscopically. On CTPV, gastric endotherapy was effective in 1 case and moderately effective in 2 cases at 1 week after treatment, with an overall effective rate of 25.00%. Additionally, the procedure was effective in 3 cases and moderately effective in 4 cases at 1 month after treatment, with an overall effective rate of 58.33%. On endoscopy, gastric endotherapy was effective in 1 case and moderately effective in 1 case at 1 week after treatment, with an overall effective rate of 16.67%. Additionally, the procedure was effective in 2 cases and moderately effective in 3 cases at 1 month after treatment, with an overall effective rate of 41.67%. CTPV was comparable to endoscopy in the therapeutic evaluation of GVs (Figure 3 and Table 4).

### 3.6. Adverse Events of Endoscopic Therapy

After endoscopic therapy, portal vein thrombosis was noted in 7 of the 24 patients (29.17%) who underwent endoscopic therapy, and no ectopic embolism was found on CTPV (Figure 3).

## 4. Discussion

Variceal hemorrhage accounts for the second most common cause of upper gastrointestinal bleeding [7]. Although endoscopy is a powerful method for GEV detection, it has limitations, such as its invasiveness and incomplete evaluation of the whole portal system. This study confirmed the feasibility of CTPV in the assessment of the vascular system, especially GEVs, in cirrhotic patients and as a less invasive method for testing the efficacy of endotherapy.

Examination by CTPV preoperatively in cirrhotic patients is necessary for better choice of treatments, as hemodynamic features vary among the different drainage pathways of GEVs [8]. CTPV with 3D images is even more accurate in drainage pathway screening than portography [9, 10]. In line with previous studies [11, 12], our study showed that CTPV is promising in the detection of the drainage pathway of GEVs and portosystemic collaterals, which lays the foundation for the evaluation of efficacy and complications after endotherapy.

Attempts have been made to use less invasive modalities as an alternative tool for the stratification of GEVs [4, 13, 14]. Spleen stiffness accurately reflects the severity of portal hypertension and is effective in EV prediction [15, 16]. A recent study developed a model with spleen stiffness assessed by point-shear-wave elastography, and it provides accurate information in cirrhotic patients for excluding endoscopy requirements [17]. However, the relationship between spleen stiffness and the EV grade remains unclear, and drainage pathways of EVs could not be determined by spleen stiffness, making it difficult to evaluate the efficacy of endotherapy by only spleen stiffness measurement. Compared to endoscopy, CTPV diminishes patients' discomfort, such as nausea, belching, and throat pain; lowers the risk of esophageal perforation, aspiration, and iatrogenic bleeding; and, more importantly, decreases the chance for hepatic encephalopathy secondary to sedation in some patients. Thus, CTPV could be considered a less invasive approach. In addition, CTPV is more cost-effective than endoscopy, and patients prefer CTPV over endoscopy [18].

The EV grade and GV type are essential for the appropriate management and prophylaxis of variceal hemorrhage. GOV1 varices that are <2 cm in diameter can be treated with EVL [19]. Endoscopic injection sclerotherapy (EIS) can be performed for GOV1 varices; however, it cannot be used to treat GOV2 and IGV1 varices, which are large and frequently associated with gastrorenal shunts [20]. CT showed high sensitivity and specificity in the detection of large GVs and was comparable to endoscopy for discriminating large varices from small varices; however, it showed unsatisfactory specificity in the detection of EVs with CTPV [18]. We found a high sensitivity (96.7% for EVs and 93.1% for GVs) and an acceptable specificity (66.7% and 75%, respectively) in the detection of GEVs. The kappa values were 0.63 and 0.62 in the detection of EVs and GVs, respectively, between CTPV and endoscopy, indicating substantial agreement. The agreement between CTPV and endoscopy in the determination of the grades and in the classification of GEVs was assessed, and the results showed substantial agreement (kappa values: 0.68 and 0.75, respectively). The difference in the results of these two techniques can be explained by several factors. During endoscopic procedures, the digestive tract was often insufflated, which changed the distorted state of the varices, while CTPV was performed without insufflation. Both procedures have difficulties in distinguishing small varices from normal rugae or mucosal folds, especially in cases of hypertensive gastropathy. Additionally, the detection and classification of GEVs may be influenced by breath holding during CTPV, which can partly decompress the varices. In the evaluation of GEVs, accuracy is greater with CT than with endoscopy, and CT is superior in assessing extraluminal pathology [21].

Studies have proven the effectiveness of CTPV in the evaluation of operations [22], but few studies have explored its evaluative role in endoscopic treatments. We devised a new evaluation system for GEV endotherapy using CTPV, and it was comparable to that for endoscopy. This system considers parameters that have been demonstrated to be associated with clinical outcomes, such as the size of residual varices and its feeding vessels after management [5, 6, 23]. Using the newly established system, we found that the overall efficacy of esophageal variceal treatments was higher when evaluated with CTPV than with endoscopy at 1 week (37.50% vs. 12.50%) and at 1 month (62.50% vs. 25.00%) after treatment, and the same trend was found in the assessment of gastric variceal treatments (25.00% vs. 16.67% at 1 week; 58.33% vs. 41.67% at 1 month). No significant difference was found between these two methods in efficacy assessment, indicating alternative potency of CTPV in GEV evaluation. In addition, patients in this study had recent hemorrhage from high-risk varices; therefore, the short-term efficacy was relatively low, and serial endotherapy is required to achieve variceal obliteration. Minor adverse events (mild dysphagia, chest pain, and transient fever) were noted postoperatively. Portal thrombosis was noted in 7 patients on CTPV, and no systemic embolization was found. Interestingly, attenuation of portal thrombosis occurred in one case, possibly due to the improvement in blood supply after endotherapy.

Postcontrast kidney injury (PC-AKI) is challenging for cirrhotic patients who undergo CTPV. There are debates concerning the causal relationship between intravenous contrast medium administration and PC-AKI in cirrhotic patients [24]. The only prospective randomized trial enrolled 91 cirrhotic patients with either ascites or renal failure, and their renal function did not show a significant difference 48 hours after contrast media administration, suggesting that cirrhosis is not a risk factor for PC-AKI [25]. Although PC-AKI is rarely seen in cirrhosis patients in clinical practice, further studies with larger samples and more centers are required before a conclusion can finally be drawn, and attention still should be paid to the renal function of patients with end-stage liver cirrhosis after CTPV. The other risk for CTPV is hypersensitivity to iodinated contrast media, with an incidence of 0.05%-0.1% [26]. But no study has demonstrated if this risk will increase in liver disease patients.

The strengths of this study are that this less invasive evaluation method provides objective, repeatable, and comprehensive assessment of GEVs on the basis of reliable agreement between CTPV and endoscopy confirmed previously. Limitations of the study include the limited number of enrolled subjects, and only the short-term efficacy of endotherapy was evaluated.

## 5. Conclusions

CTPV offers a less invasive and reliable alternative to endoscopy for the detection and classification of GEVs. Our evaluation system might be appropriate to assess the efficacy of endotherapy for GEVs. Our findings suggest the possibility of reduced stress for endoscopists and improved compliance of cirrhotic patients with GEVs regarding return visits by using CTPV.

## Figures and Tables

**Table 1:** Diagnostic performance of CTPV in determining the grades of esophageal varices.

Endoscopic findings	CTPV findings				
	Negative	Mild	Moderate	Severe	Total
Negative	2	1	0	0	3
Mild	0	2	1	0	3
Moderate	1	1	4	1	7
Severe	0	0	1	19	20
Total	3	4	6	20	33

*P* = 0.01, kappa = 0.68; CTPV: computed tomography portal venography.

**Table 2:** Diagnostic performance of CTPV in the classification of gastric varices.

Endoscopic findings	CTPV findings					
	Negative	GOV1	GOV2	IGV1	IGV2	Total
Negative	0	0	0	0	0	0
GOV1	1	14	2	0	0	17
GOV2	0	1	13	0	0	14
IGV1	0	0	1	1	0	2
IGV2	0	0	0	0	0	0
Total	1	15	16	1	0	33

*P* = 0.01, kappa = 0.75; CTPV: computed tomography portal venography; GOV: gastroesophageal varices; IGV: isolated gastric varices.

**Table 3:** Collateral circulations in cirrhotic patients on computed tomography portal venography.

Veins	Number (total = 33)	Percentage (%)
Portosystemic collaterals		
Esophageal varices	30	90.91
Gastric varices	28	84.85
Paraesophageal varices	21	63.64
Abdominal wall varices	5	15.15
Paravertebral varices	2	6.06
Duodenal varices	1	3.03
Afferent veins of esophageal and gastric varices		
Left gastric vein	13	39.39
Short/posterior gastric vein	4	12.12
Left gastric vein+short/posterior gastric vein	16	48.48
Efferent veins of esophageal and gastric varices		
Azygos vein	15	45.46
Gastric/splenorenal shunt	5	15.15
Azygos+gastric/splenorenal shunt	13	39.39

**Table 4:** The efficacy of endotherapy in the management of gastroesophageal varices evaluated with CTPV and endoscopy.

Location of varices	Method	Time	Effective (*n*)	Moderately effective (*n*)	Ineffective (*n*)	Efficacy (%)	*P* value
Esophagus	Endoscopy	1 week after treatment	0	2	14	12.50	0.22
	CTPV		0	6	10	37.50	
	Endoscopy	1 month after treatment	1	3	12	25.00	0.07
	CTPV		2	8	6	62.50	

Stomach	Endoscopy	1 week after treatment	1	1	10	16.67	1
	CTPV		1	2	9	25.00	
	Endoscopy	1 month after treatment	2	3	7	41.67	0.68
	CTPV		3	4	5	58.33	

CTPV: computed tomography portal venography.

## Data Availability

The data used to support the findings of this study are available from the corresponding author upon request.

## Supplementary Materials

Supplementary Materials The gastric varix type, esophageal varix grade, and some results are shown. Table S1: baseline characteristics of the patients. Table S2: diagnostic performance of computed tomography portal venography (CTPV) in identifying esophageal and gastric varices.

## References

[1] Zanetto A., Garcia-Tsao G. (2019). Management of acute variceal hemorrhage. *F1000Res*.

[2] Chinese Society of Surgery, Chinese Medical Association, Portal Hypertension Surgery (2019). Expert consensus on diagnosis and treatment of esophagogastric variceal bleeding in cirrhotic portal hypertension (2019 edition). *Chinese Journal of Surgery*.

[3] Bandali M. F., Mirakhur A., Lee E. W. (2017). Portal hypertension: imaging of portosystemic collateral pathways and associated image-guided therapy. *World Journal of Gastroenterology*.

[4] Li Q., Wang R., Guo X. (2019). Contrast-enhanced CT may be a diagnostic alternative for gastroesophageal varices in cirrhosis with and without previous endoscopic variceal therapy. *Gastroenterology Research and Practice*.

[5] Lee H. A., Goh H. G., Kim T. H. (2020). Evaluation of treatment response after endoscopic variceal obturation with abdominal computed tomography. *Gut and Liver*.

[6] Rice J. P., Lubner M., Taylor A. (2011). CT portography with gastric variceal volume measurements in the evaluation of endoscopic therapeutic efficacy of tissue adhesive injection into gastric varices: a pilot study. *Digestive Diseases and Sciences*.

[7] Kamboj A. K., Hoversten P., Leggett C. L. (2019). Upper gastrointestinal bleeding: etiologies and management. *Mayo Clinic Proceedings*.

[8] Gaba R. C., Couture P. M., Lakhoo J. (2015). Gastroesophageal variceal filling and drainage pathways: an angiographic description of afferent and efferent venous anatomic patterns. *J Clin Imaging Sci*.

[9] Zhu K., Meng X., Pang P. (2010). Gastric varices in patients with portal hypertension: evaluation with multidetector row CT. *Journal of Clinical Gastroenterology*.

[10] Ishikawa T., Ushiki T., Mizuno K. (2005). CT-maximum intensity projection is a clinically useful modality for the detection of gastric varices. *World Journal of Gastroenterology*.

[11] Kawanaka H., Akahoshi T., Nagao Y. (2018). Customization of laparoscopic gastric devascularization and splenectomy for gastric varices based on CT vascular anatomy. *Surgical Endoscopy*.

[12] Matsumoto Y., Hidaka H., Matsunaga K. (2016). Three-dimensional computed tomography of portopulmonary venous anastomoses in patients with esophageal varices before treatment. *Hepatology Research : The Official Journal of the Japan Society of Hepatology*.

[13] Kihira S., Kagen A. C., Vasudevan P. (2016). Non-invasive prediction of portal pressures using CT and MRI in chronic liver disease. *Abdominal Radiology*.

[14] Elalfy H., Elsherbiny W., Abdel Rahman A. (2016). Diagnostic non-invasive model of large risky esophageal varices in cirrhotic hepatitis C virus patients. *World Journal of Hepatology*.

[15] Fierbinteanu-Braticevici C., Tribus L., Peagu R. (2019). Spleen stiffness as predictor of esophageal varices in cirrhosis of different etiologies. *Scientific Reports*.

[16] Berzigotti A. (2017). Non-invasive evaluation of portal hypertension using ultrasound elastography. *Journal of Hepatology*.

[17] Giuffrè M., Macor D., Masutti F. (2020). Spleen stiffness probability index (SSPI): a simple and accurate method to detect esophageal varices in patients with compensated liver cirrhosis. *Annals of Hepatology*.

[18] Perri R. E., Chiorean M. V., Fidler J. L. (2008). A prospective evaluation of computerized tomographic (CT) scanning as a screening modality for esophageal varices. *Hepatology*.

[19] Sarin S. K. (1997). Long-term follow-up of gastric variceal sclerotherapy: an eleven-year experience. *Gastrointestinal Endoscopy*.

[20] Sarin S. K., Kumar A. (2014). Endoscopic treatment of gastric varices. *Clinics in Liver Disease*.

[21] Hassan M., Husen Y., Abbasi S. U., Hussain Z. (2019). Diagnostic accuracy of multidetector computed tomography in detection of esophageal varices. *Cureus*.

[22] Turk Y., Salmaslioglu A., Sasani H. (2019). The role of multislice computerized tomography angiography in assessing postoperative vascular complications in liver transplant patients. *Turkish Journal of Medical Sciences*.

[23] Kodama H., Aikata H., Takaki S. (2010). Evaluation of portosystemic collaterals by MDCT-MPR imaging for management of hemorrhagic esophageal varices. *European Journal of Radiology*.

[24] Safi W., Rauscher I., Umgelter A. (2015). Contrast-induced acute kidney injury in cirrhotic patients. A retrospective analysis. *Annals of Hepatology*.

[25] Guevara M., Fernández-Esparrach G., Alessandria C. (2004). Effects of contrast media on renal function in patients with cirrhosis: a prospective study. *Hepatology*.

[26] Rosado Ingelmo A., Doña Diaz I., Cabañas Moreno R. (2016). Clinical practice guidelines for diagnosis and management of hypersensitivity reactions to contrast media. *Journal of Investigational Allergology and Clinical Immunology*.

