# Supplementary material for: Efficacy of CTPV for Diagnostic and Therapeutic Assessment: Comparison with Endoscopy in Cirrhotic Patients with Gastroesophageal Varices
Source: Gastroenterol Res Pract. 2020 Jun 5;2020:6268570. doi: 10.1155/2020/6268570 (PMC7293743; doi:10.1155/2020/6268570)
Supplement: Supplementary Materials — The gastric varix type, esophageal varix grade, and some results are shown. Table S1: baseline characteristics of the patients. Table S2: diagnostic performance of computed tomography portal venography (CTPV) in identifying esophageal and gastric varices. [file 6268570.f1.pdf]

Supplementary Methods

The types of gastric varices (GVs) were defined according to the Sarin classification system[1] as follows: GOV1 (type one gastroesophageal varices) is continuous with esophageal varices (EVs) and located along the lesser curvature of the stomach; GOV2 (type two gastroesophageal varices) extends along the greater curvature to the gastric fundus; isolated GV (IGV) 1 is located alone in the fundus; IGV2 refers to ectopic varices located anywhere in the stomach and duodenum, except the fundus. EVs on CTPV were graded using a modified system proposed by Kim et al.[2] as follows: mild, discrete enhancing lesions on the luminal surface of the esophageal wall without protrusion or slight protrusion inward, with a diameter < 5 mm; moderate, tortuous enhancing variceal columns occupying no more than half of the esophageal lumen, with a diameter < 5 mm; and severe, several notable and coiled lesions protruding into the esophageal lumen, with a diameter > 5 mm or varices occupying more than half of the esophageal lumen. A finding of circumferential esophageal or gastric wall thickening alone without any nodular enhancing lesions was not considered to be varices. EVs on endoscopy were graded as follows[3]: mild, varices run straight or slightly tortuous, with no red color sign; moderate, varices run straight or slightly tortuous with a red color sign or those with a serpent-form appearance with no red color sign; and severe, serpent-form appearance with a red color sign or varices which are tortuous and with a nodular appearance or tumor-like appearance. Two reviewers evaluated the EV grade and the GV type by consensus.

Supplementary Tables

Table S1. Baseline characteristics of the patients

Characteristic	No. of patients
Sex	
Male	21
Female	12
Cause of cirrhosis	
HBV	17
HCV	6
Alcohol	2
Drugs	1
PBC	3
Others	4
Child-Pugh classification	
Class A	5
Class B	11
Class C	17
Endoscopic treatment of esophageal varices	
EVL	11
EIS	4
Tissue adhesive injection	1
Endoscopic treatment of gastric varices	
Tissue adhesive injection	10
EVL	2

HBV, hepatitis B virus; HCV, hepatitis C virus; PBC, primary biliary cholangitis; EVL, endoscopic variceal ligation; EIS, endoscopic injection sclerotherapy

Table S2. Diagnostic performance of computed tomography portal venography (CTPV) in identifying esophageal and gastric varices

		Endoscopy(+) varices	Endoscopy(−) varices
EVs**†	CTPV(+) varices	29	1
	CTPV(−) varices	1	2
GVs**‡	CTPV(+) varices	27	1
	CTPV(−) varices	2	3

**† *P*=0.01, kappa = 0.63; **‡ *P*=0.01, kappa = 0.62.

EVs: esophageal varices; GVs: gastric varices

Reference

1.S. K. Sarin, D. Lahoti, S. P. Saxena et al., "Prevalence, classification and natural history of gastric varices: a long-term follow-up study in 568 portal hypertension patients," Hepatology, vol.16, no. 6, pp. 1343-1349, 1992.

2.Y. J. Kim, S. S. Raman, N. C. Yu et al., "Esophageal varices in cirrhotic patients: evaluation with liver CT," AJR American journal of roentgenology, vol.188, no. 1, pp. 139-144, 2007.

3.Chinese Society of Endoscopy, Chinese Medical Association, "Expert consensus on diagnosis and treatment of esophagogastric variceal bleeding in cirrhotic portal hypertension (2015 edition)," Chinese Journal of Practical Surgery, vol.35, no. 10, pp. 1086-1090, 2015.
